# A Two-Hit Model of The Biological Origin of Posttraumatic Stress Disorder (PTSD)

**Published:** 2018-10-01

**Authors:** Apostolos P. Georgopoulos, Lisa M. James, Peka Christova, Brian E. Engdahl

**Affiliations:** 1 Department of Veterans Affairs Health Care System, Brain Sciences Center Minneapolis, Minnesota, USA; 2 Department of Neuroscience, University of Minnesota Medical School, Minneapolis, Minnesota, USA; 3 Center for Cognitive Sciences, University of Minnesota, Minneapolis, Minnesota, USA; 4 Department of Psychiatry, University of Minnesota Medical School, Minneapolis, Minnesota, USA; 5 Department of Neurology, University of Minnesota Medical School, Minneapolis, Minnesota, USA; 6 Department of Psychology, University of Minnesota Medical School, Minneapolis, Minnesota, USA

**Keywords:** Posttraumatic stress disorder (PTSD), Intercellular adhesion molecule 5 (ICAM-5), Glutamatergic neurotransmission, Persistent antigen, Functional magnetic resonance imaging (fMRI), Magnetoencephalography, Gulf War Illness

## Abstract

Posttraumatic stress disorder (PTSD) is a debilitating disorder that can develop following exposure to a traumatic event. Although the cause of PTSD is known, the brain mechanisms of its development remain unknown, especially why it arises in some people but not in others. Most of the research on PTSD has dealt with psychological and brain mechanisms underlying its symptomatology, including intrusive memories, fear and avoidance (see ref.^1^ for a broad coverage of PTSD research)^1^. Here we focus, instead, on the origin of PTSD, namely on the neural mechanisms underlying its development. Specifically, we propose a two-hit model for PTSD development, with the following components. (a) The 1st hit is a neuroimmune challenge, as a preexisting condition, and the 2nd hit is intense glutamatergic neurotransmission, induced by the traumatic event; (b) the key molecule that mediates the effects of these two hits is intercellular adhesion molecule 5 (ICAM-5) which was found to be differentially expressed in PTSD^2^. ICAM-5 is activated by neuroimmune challenge^3,4^ and glutamatergic neurotransmission^5,6^, it further enhances glutamatergic transmission^6^, and exerts a potent effect on synapse formation and neural plasticity, in addition to immunoregulatory functions^3,4,7^; and (c) with respect to the neural network(s) involved, the brain areas most involved are medial temporal cortical areas, and interconnected cortical and subcortical areas^8–10^. We hypothesize that the net result of intense glutamatergic transmission in those areas induced by a traumatic event in the presence of ongoing neuroimmune challenge leads to increased levels of ICAM-5 which further enhances glutamatergic transmission and thus leads to a state of a neural network with highly correlated neural interactions, as has been observed in functional neuroimaging studies^8–10^. We assume that such a “locked-in” network underlies the intrusive re-experiencing in PTSD and maintains associated symptomatology, such as fear and avoidance.

## Introduction

Posttraumatic stress disorder (PTSD) is a psychiatric disorder that can develop in response to exposure to traumatic events, with hallmark symptoms including intrusive recollections or re-experiencing of the traumatic event, avoidance of trauma reminders, cognitive and affective changes, and hyperarousal^11^. PTSD is associated with high rates of psychiatric and medical comorbidities^12–16^, and increased mortality^17,18^. Although 90% of individuals are exposed to potentially traumatic events, only 7–10% of the general population^19,20^ and 10–30% of veterans^21–23^, develop PTSD suggesting that trauma alone is not sufficient. Rather, the typical outcome following trauma exposure is resilience^24^. Nonetheless, for those who develop PTSD, the symptoms may lead to significant chronic functional impairment. Furthermore, although several psychological and pharmacological interventions have been shown to be effective in reducing symptoms, treatment outcomes vary considerably with many individuals failing to respond and up to two-thirds of treatment responders failing to achieve remission^25^, leading many to emphasize the need for novel interventions.

### Current Theories of PTSD

Numerous psychological and neurobiological theories of PTSD have been proposed. Psychological theories generally focus on cognitive functions associated with trauma including abnormal information processing, maladaptive interpretations about traumatic experiences, deleterious effects of trauma on belief systems, and memory disturbances, as well as affective contributions to these processes (several dominant models are reviewed in ref.^26^). Similarly, several neurobiological theories of PTSD have emerged and are summarized elsewhere^27^. Briefly, the dominant models focus on dysfunctional fear learning, threat detection, executive function/emotion regulation, and contextual processing and the associated neural regions including the amygdala, midcingulate cortex, insula, medial prefrontal cortex, and the hippocampus. These theories have been useful in explaining PTSD-associated symptomatology and have, in some cases, provided the basis for psychological interventions; however, they largely fail to adequately explain the development of PTSD. Thus, here we present a novel model of PTSD that elucidates the origin of the disorder, accounts for resilient vs pathological trajectories following trauma exposure, and provides an explanation for treatment non-response.

### Biological Correlates of PTSD

Biological correlates of PTSD have been widely investigated with numerous studies identifying physiological, neuroendocrine, and genetic factors in addition to structural and functional brain alterations (for reviews see refs.^28,29^). At the brain network level, anomalous neural interactions characterized by hypercorrelation^9^ have been shown to distinguish PTSD from controls^8,10^, with both cortical^9^ and limbic regions involved^30^. Based on those findings, we hypothesized that the persistence of highly correlated neural ensembles underlies characteristic PTSD symptoms^30^. Here we discuss the role of immune system involvement in facilitating the persistent hypercorrelated network associated with PTSD.

Immunological involvement in PTSD is a promising avenue that has recently emerged. Recent reviews^29,31^ note immune system alterations including increased levels of pro-inflammatory cytokines, C-reactive protein (CRP), and tumor necrosis factor (TNF)-α in PTSD. Notably, cytokines have been found to have profound effects on the HPA axis^32^, which is intimately linked to PTSD. In addition, FKBP5, a gene involved in immune regulation and a modulator of HPA axis function have been implicated in PTSD^33^. One particularly noteworthy study demonstrated that pre-deployment CRP predicted post-deployment PTSD^34^, suggesting inflammation may be a predisposing factor for PTSD. To that end, it has been speculated that inflammation resulting from traumatic brain injury underlies the high rates of co-occurrence of brain injury and PTSD^35^. Finally, changes in inflammatory markers have been reported following PTSD treatment^31^, and administration of hydrocortisone, an anti-inflammatory agent, immediately after trauma exposure has been shown to prevent PTSD in human subjects and promote dendrite growth and spine density in stressed animals^36^. In fact, those latter positive neural changes extended to other domains as well, including increased levels of brain-derived neurotrophic factor. These findings underscore the intricate interplay among neural, hormonal and inflammatory factors in serious stress and the possible alleviation of its deleterious effects.

### Biological Basis of the Origin of PTSD: A Two-Hit Model

PTSD is typically initiated by massive multi-sensory activation produced by the trauma exposure^30^. The layout of cortical connectivity, as delineated by Jones and Powell^37^, channels the flow of activation from primary cortical sensory areas, through intermediate interconnected stations, to ultimate convergence in the parieto-temporal and frontal cortices, and therefrom distribution to rostral and medial temporal areas, anterior and orbitofrontal frontal areas, and subcortical areas^38^. We have proposed that these multiple and converging interactions induce stable correlations among the various areas of convergence^30^, an idea supported by the results of modeling studies^30^. Such stable correlations have been observed in PTSD^9^, in contrast to healthy controls where a systematic reduction in trauma-related neural correlations was observed^9^. We have hypothesized that this stable correlation pattern represents a “locked-in” state of the late-stage sensory processing that underlies the occurrence of intrusive traumatic re-experiences and flashbacks^8^. It is reasonable to suppose that trauma exposure will initially induce such correlations to all brains; the question then is: why such correlations would persist in PTSD with all the untoward consequences? The considerations above and the findings of recent studies led us to propose a two-hit model for PTSD development, with the following components. (i) The 1^st^ hit is neuroimmune challenge, as a preexisting condition; (ii) the 2^nd^ hit is intense glutamatergic neurotransmission, induced by the traumatic event; (iii) the key molecule that mediates the effects of these two hits is intercellular adhesion molecule 5 (ICAM-5) which was found to be differentially expressed in PTSD^2^ (it is very possible that other molecules, in addition to ICAM-5, might be involved in this process); and (iv) the net result is a hypercorrelated state among higher-order association cortical and subcortical areas, with a focus on the temporal lobe^8–10^. Specifically, we hypothesize that the net result of intense glutamatergic transmission in those areas induced by a traumatic event in the presence of ongoing neuroimmune challenge leads to increased levels of ICAM-5 which further enhances glutamatergic transmission and thus leads to a state of a neural network with highly correlated neural interactions, as has been observed in functional neuroimaging studies^8–10^. We assume that such a “locked-in” network underlies the intrusive re-experiencing in PTSD and maintains associated symptomatology, such as fear and avoidance.

### The Role of InterCellular Adhesion Molecule 5 (ICAM-5)

A recent study using transcriptomic analysis immediately following exposure to a traumatic event found that ICAM-5 was differentially expressed in people who subsequently developed PTSD following that exposure^2^. This finding plays a central role in our model, based on the dual role of this molecule, namely in glutamatergic neurotransmission^5,6^ and local immunosuppression^3,4^. ICAM-5 is a member of the immunoglobulin superfamily, a group of adhesive glycoproteins expressed on cell surfaces that are primarily involved in cellular processes underlying the immune system and inflammation. ICAM-5, however, is unique in that it is the only ICAM that is expressed exclusively in the brain^39^, and it has been shown to play a critical role in dendritic development, synapse maturation, and associated neural network plasticity, in addition to typical ICAM immunoregulatory functions^3–7^. Moreover, ICAM-5 expression is limited to neurons in select telencephalic regions, including the neocortex, hippocampus, amygdala, caudate nucleus, and putamen, areas that are involved in higher-order functions such as learning and memory. For biological action, ICAM-5 is cleaved resulting in soluble ICAM-5 (sICAM-5); sICAM-5 enhances glutamatergic neurotransmission^6^ and exerts local immunosuppression relating to both innate and adaptive immunity^3,4^. Both of these actions bear directly on our hypothesis regarding its role in inducing and maintaining PTSD’s intrusive memory and traumatic re-experiencing symptomatology.

ICAM-5 exerts an immunosuppressive action, as mentioned above, in response to antigenic challenges. sICAM-5 is released in response to viral infections, including encephalitis of diverse causes^40^. Its role helps keep local inflammation at low levels. Therefore, it is reasonable to suppose that sICAM-5 will be produced in response to local antigenic challenges. According to our model, it is the occurrence of a traumatic event in such a background that is crucial for PTSD development.

### Glutamatergic Neurotransmission Enhances sICAM-5 production, and Vice Versa

In the realm of synaptic transmission, independently of antigenic challenge, the key factor producing ICAM-5 cleavage, and hence production of sICAM-5, is activation of glutamate receptors, specifically N-methyl-D- aspartate (NMDA)^5^ and α-amino-3-hydroxy-5-methyl-4- isoxazolepropionic acid (AMPA) receptors^6^. sICAM-5, in turn, enhances glutamatergic transmission^6^, thus creating a vicious cycle of an activated network. Since the dominant, if not exclusive, excitatory neurotransmission in the cerebral cortex is glutamatergic, it follows that the massive neuronal activity initiated by a traumatic event will lead to increased levels of sICAM-5, which, in turn, will further enhance glutamatergic transmission. We hypothesize that this results in “fixing” those synapses, which, at the network level, would represent the memorized experience of the traumatic event. If that were universally the case for all people, why does PTSD develop in some but not others? For this, we postulate that the local level/concentration of sICAM-5 is the crucial factor: the higher the sICAM-5 level, the stronger the synaptic “tightness”, and the associated correlations. This would explain why more serious traumatic events would be likely to have more lasting consequences but could not explain how traumatic events of similar intensity would lead to PTSD in some but not others. For this, we postulate a second hit, as follows.

### PTSD: Intense Glutamatergic Transmission in The Background of Immune Challenge

We postulate that intense glutamate transmission induced by a major traumatic event occurring in the background of ongoing antigenic/immunological challenge will lead to a large increase of sICAM-5 leading, in turn, to substantially enhanced synaptic maturation. We further postulate that this essentially “fixes” the synaptic landscape of the network activated by the traumatic event. As mentioned above, this network mainly comprises anterior and medial temporal areas, anterior frontal and orbitofrontal areas, and subcortical limbic areas^8–10^. In this context, we explain the observed relation between ICAM-5 production and PTSD development^2^ by hypothesizing that ICAM-5 production was a pre-existing condition due to inflammatory/immunogenic challenge on the background of which the massive glutamate input induced a synaptic “locking” of the activated network, manifested behaviorally as persistent re-experiencing of the traumatic event. This interpretation is consistent with the findings of 3 studies, namely (a) that an elevated blood CRP is predictive of PTSD development^34^, (b) that intravenous (IV) injection of hydrocortisone (an anti-inflammatory agent) shortly after the occurrence of the traumatic event reduced the chance of PTSD development^36^, and (c) that IV injection of ketamine (a noncompetitive NMDA antagonist with relatively selective action in the midcingulate cortex^41^) in chronic PTSD alleviates the severity of its symptoms^42^. This last study, in particular, is in keeping with our hypothesis that a persisting, enhanced glutamatergic transmission underlies PTSD symptomatology.

### Background Immune Challenge: Persistent Antigens and PTSD

Our two-hit model for PTSD development predicts that exposure to trauma in the presence of neuroimmune challenge would increase their risk of developing PTSD. As mentioned above, the presence of inflammation, as indicated by increased plasma CRP, is a risk factor for developing PTSD following trauma exposure^34^ and, conversely, the presence of PTSD is a risk factor for subsequently developing an autoimmune disease^43,44^. Moreover, we found in a recent study^45^ that Gulf War Illness (GWI), an immune-related disorder^46–48^, primes the occurrence of PTSD.

The various mechanisms underlying the interplay between PTSD and associated immune-related comorbidities, and the relevant neuroendocrine alterations involved, have been discussed succinctly^49^. In general, it is not clear which is the primary cause. It is very possible that PTSD and immune-related disorders share common functional alterations, predisposing for each other, that will need further research to be disentangled. Based on our initial findings of reduced Human Leukocyte Antigen (HLA) class 2 protection in GWI^46^, and on our more recent finding that HLA allele DRB1*13:02 prevents brain atrophy in GWI^48^, we proposed^48^ that an important underlying factor for GWI consists of “persistent antigens”, that is, antigens (of various kinds) to which veterans of the 1991 Gulf War were exposed (most probably through the vaccines received) but which could not be eliminated due the lack of HLA protection^46^. This hypothesis is in keeping with the results of our study^51^ that blood serum from veterans suffering from GWI exerts detrimental effects on brain cultures that are prevented by the addition of serum from healthy Gulf War era veterans. Finally, the link between the “persistent antigen” hypothesis^47^ and immune-related disorders is reinforced by the protection conferred by DRB1*13:02 on both GWI^47^ and a broad range of typical autoimmune diseases^50^. Thus, the underlying immune challenge we propose in our two-hit PTSD model here could be attributed to persistent antigens that would reflect reduced adaptive immunity protection in susceptive individuals. This issue remains to be clarified by an extensive investigation of HLA makeup in PTSD. In addition, the nature of the hypothesized persistent antigens could be investigated by experiments employing neural cultures^51^. Using such cultures, we have shown that deleterious effects of persistent antigens circulating in the blood can be reduced or eliminated^51^; this opens the possibility of treating PTSD by identifying and eliminating persistent antigens that otherwise hinder neural plasticity and recovery.

## Figures and Tables

**Figure 1.**
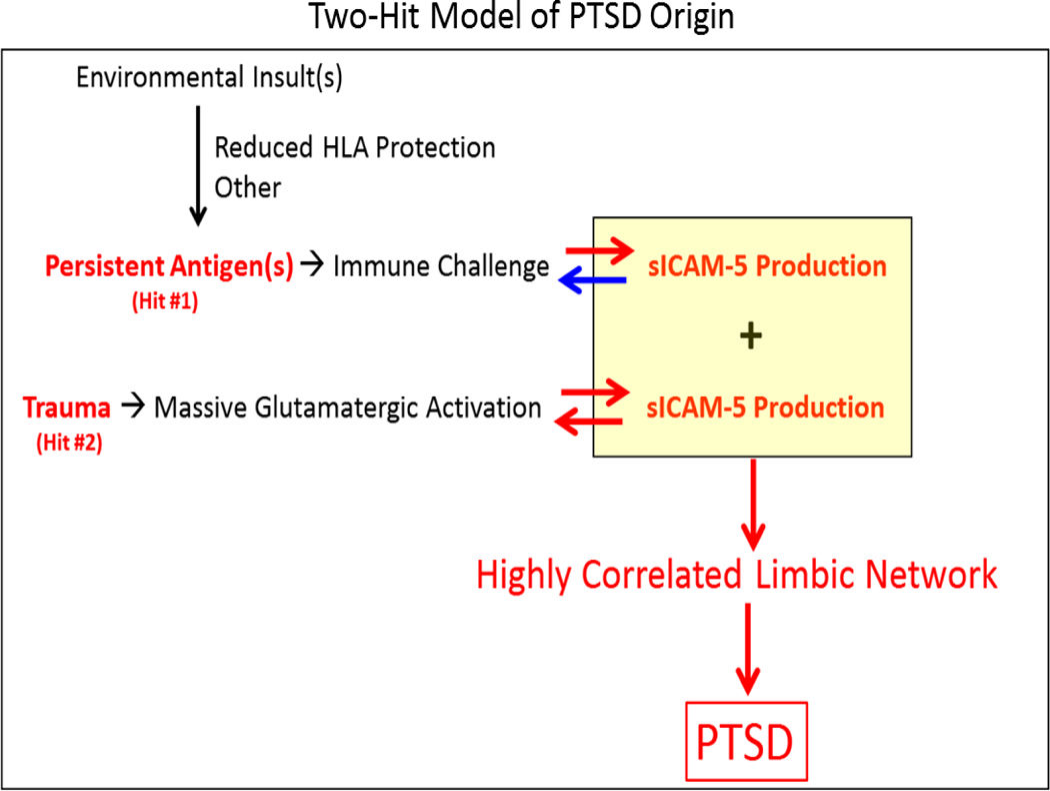
Schematic diagram of the two-hit model of PTSD origin. Red arrows indicate enhancing effect; blue arrow indicates immunosuppressive effect. See text for details.
